# Risk of Invasive Meningococcal Disease in Children Aged <11 Years in the United States

**DOI:** 10.1093/ofid/ofag185

**Published:** 2026-04-10

**Authors:** Oscar Herrera-Restrepo, Elizabeth Packnett, Megan K Richards, Elise Kuylen, Tosin O Olaiya, Thatiana Pinto, Lindsay C Landgrave, Ryan Ross, Andrew G Allmon

**Affiliations:** GSK, Philadelphia, Pennsylvania, USA; Merative, Ann Arbor, Michigan, USA; Merative, Ann Arbor, Michigan, USA; GSK, Wavre, Belgium; GSK, Philadelphia, Pennsylvania, USA; GSK, Wavre, Belgium; GSK, Philadelphia, Pennsylvania, USA; Merative, Ann Arbor, Michigan, USA; GSK, Durham, North Carolina, USA

**Keywords:** IMD risk factors, incidence rate, invasive meningococcal disease, pediatric

## Abstract

**Background:**

Invasive meningococcal disease (IMD) is associated with high case-fatality rates and severe long-term sequelae. In the United States (US), IMD incidence rate (IR) is highest among infants aged <1 year, with most cases occurring in infants aged <6 months. This study estimated IMD incidence in commercially and Medicaid-insured infants, toddlers, and children aged <11 years.

**Methods:**

This retrospective claims analysis utilized real-world data (01 January 2005 to 31 December 2022) from the MarketScan® Commercial and Multi-State Medicaid Research Databases of infants (aged <1 year), toddlers (aged 1–4 years), and children (aged 5–10 years). IMD incidence, time at risk for IMD, IR, and IR ratio (IRR) were evaluated overall and by demographic/clinical characteristics.

**Results:**

Within the commercially and Medicaid-insured cohorts, 21 and 115 infants, 34 and 80 toddlers, and 36 and 37 children with IMD were identified, respectively. Overall IMD IRs in infants, toddlers, and children were 0.45, 0.17, and 0.11 per 100 000 person-years (PY; commercially insured) and 1.97, 0.45, and 0.18 per 100 000 PY (Medicaid-insured), respectively. Incidence rates were highest in infants aged <4 months (1.03 and 4.65 per 100 000 PY for infants aged 2 to <3 months [commercially insured] and 1 to <2 months [Medicaid-insured], respectively). Significantly elevated IRRs were observed among infants and children with specific demographic and clinical characteristics, including residence in rural counties, immunocompromised status, and complement component deficiency.

**Conclusions:**

IMD incidence is low in the United States, but varies by age, insurance type, and demographic/clinical characteristics. Timely preventative interventions, such as early vaccination, may reduce the incidence, risk, and associated burden of IMD.

## Teaser

In this retrospective analysis of medical claims, incidence of invasive meningococcal disease (IMD) among United States infants, toddlers, and children was characterized by age, insurance type, and demographic/clinical characteristics. IMD incidence was highest in infants, particularly those aged <4 months, and overall incidence was higher in Medicaid-insured versus commercially-insured individuals regardless of age, highlighting opportunities for early preventive measures to reduce IMD burden.

## Graphical Abstract

**Graphical Abstract:**
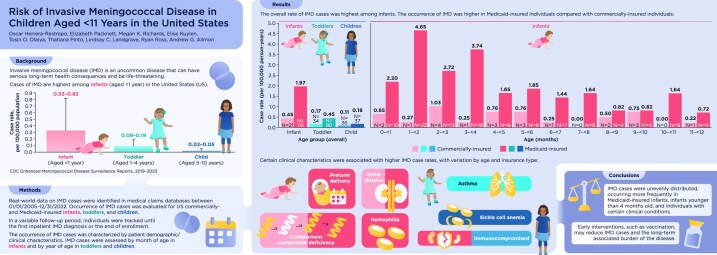

Invasive meningococcal disease (IMD), caused by the bacterium *Neisseria meningitidis*, is uncommon yet serious and potentially life-threatening, with sudden onset and rapid progression [1, 2]. The disease is associated with high case-fatality rates (10%–15%, even when treated with appropriate antibiotic therapy) and severe long-term sequelae such as limb amputation and neurologic deficits occur in up to 40% of survivors [1–4].

Globally, most IMD cases are caused by 6 meningococcal serogroups (A, B, C, W, X, and Y) [5]. In the United States (US), there are 2 quadrivalent conjugate vaccines to protect against IMD caused by serogroups A, C, W, and Y (MenACWY) [6]. At the time of the study, the Centers for Disease Control and Prevention (CDC) routinely recommended MenACWY for individuals aged 11–12 years, with a booster dose at age 16 years, and for individuals aged ≥2 months at increased risk for meningococcal disease [7]; on January 5, 2026, the CDC recommendations for MenACWY doses at ages 11–12 years and 16 years were changed from a routine recommendation to shared clinical decision-making (SCDM) [8, 9]. Two vaccines to protect against IMD caused by serogroup B (MenB) are recommended under SCDM for individuals aged 16–23 years and routinely for those aged ≥10 years at increased risk for meningococcal disease (those with anatomic or functional asplenia, complement component deficiency, complement inhibitor use, microbiologists with routine exposure to *N. meningitidis* isolates, or persons at increased risk during an outbreak) [6]. Finally, 2 vaccines are approved in the United States against serogroups A, B, C, W, and Y (MenABCWY); 1 dose of MenABCWY may be used in place of separate doses of MenACWY and MenB when both MenACWY and MenB vaccines are indicated at the same visit [6]. Despite the availability of well-tolerated and effective vaccines, no MenB-containing vaccine is currently approved for children aged <10 years in the United States, regardless of risk [3, 5].

In the United States, IMD incidence sharply increased in 2022 after steadily decreasing from 2006 to 2021; however, incidence rates (IRs) have consistently remained highest among infants aged <1 year, primarily due to serogroup B infections [10–12]. From 2019 to 2023, between 35 and 68 confirmed and probable cases of IMD were identified each year in children aged <11 years, with IRs of 0.33–0.82, 0.09–0.19, and 0.02–0.05 per 100 000 population in individuals aged <1 year, 1–4 years, and 5–10 years, respectively [10, 13–16]. Despite research on IMD outbreaks in adolescents and young adults, risk factors for IMD infection among infants and young children remain less understood [2, 7, 17, 18]. A study evaluating the epidemiology of meningococcal disease in US infants from 2006 to 2012 found that the IMD incidence in infants varied by month of age [19]. However, IMD incidence in infants has not been well characterized by month of age, limiting our understanding of the evolution of IMD risk over the first year of life [10, 14, 20].

Prior studies from 2010 and 2015 using the CDC Active Bacterial Core (ABC) surveillance data estimated IMD incidence by month in infants; most cases occurred in infants aged <6 months, with implications for future vaccination programs [19, 21]. However, these studies were limited to ABC sites and not the entire US population; furthermore, incidence was not estimated by demographic/clinical characteristics [19, 21]. A more comprehensive and up-to-date understanding of IMD incidence in individuals aged <11 years may support the development of public health policy to prevent IMD in this population. This study aimed to estimate IMD incidence in US commercially and Medicaid-insured infants, toddlers, and children, overall and by month of age and patient demographics/clinical characteristics.

## METHODS

### Study Design and Populations

This retrospective analysis utilized real-world data from the MarketScan® Commercial and Multi-State Medicaid Research Databases (01 July 2004 to 31 December 2022) to estimate IMD incidence in US commercially and Medicaid-insured infants, toddlers, and children. The MarketScan Commercial Database is a convenience sample of large employers and health plans across the United States that includes a variety of fee-for-service, preferred provider organizations, and capitated health plans. The MarketScan Multi-State Medicaid Database reflects healthcare service use of Medicaid enrollees. MarketScan National Weights was used to estimate IMD incidence for individuals with commercial employer-sponsored insurance (ESI).

Children aged <11 years were identified in the MarketScan Research Databases between 01 January 2005 and 31 December 2022. The eligibility timeframe aligned with MenACWY vaccine availability in the United States (2005) through the last full year of available data when this study began. In both insurance cohorts, patients with IMD were identified as those with a diagnosis code for IMD (International Classification of Diseases [ICD]-9: 036.X; ICD-10: A39.X) in the primary position on an inpatient claim, consistent with recent research on IMD incidence using administrative claims databases [20, 22]. Three age cohorts (infants aged <1 year, toddlers aged 1–4 years, and children aged 5–10 years during the study period) were identified using exact date of birth. Individuals could be included in more than 1 cohort and index dates were assigned based on the first date of enrollment in each age cohort. Those with medical claims indicating that they were tested for IMD prior to index date, but without an associated diagnosis claim for IMD, were included in the study, as a claim for IMD testing without an associated diagnosis claim for IMD was not considered an IMD case. Individuals with ≥1 nondiagnostic or nonrule-out medical claim for IMD prior to index date, those with an IMD diagnosis on an outpatient claim unrelated to IMD testing, and those with inpatient claims with IMD diagnosis in a nonprimary position prior to the index date were excluded.

**Figure 1.**
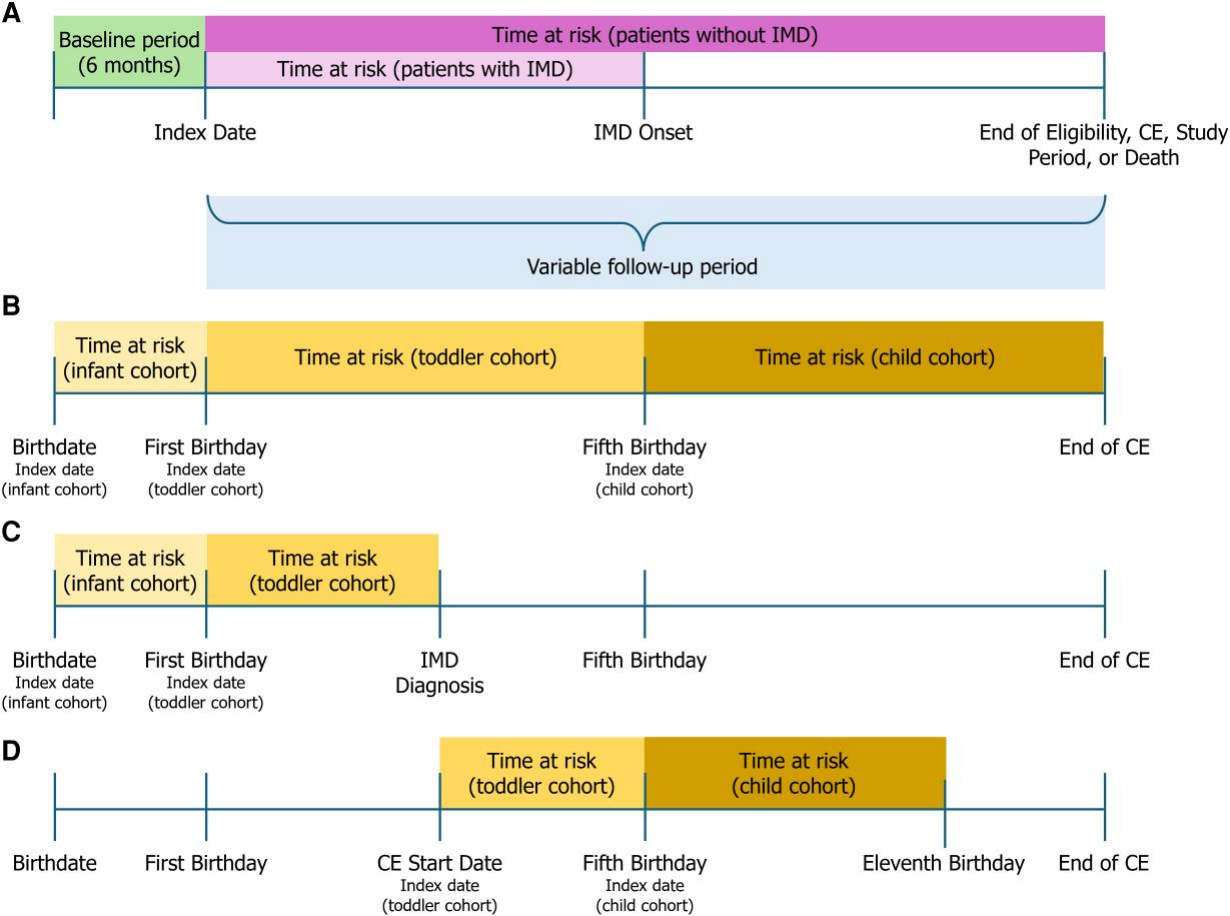
*A*, Overall study design schematic. *B*, Scenario where individual was continuously enrolled from birthdate to age 9 y and does not have a claim for IMD. *C*, Scenario where individual was continuously enrolled from birthdate to age 9 y and has a claim for IMD at age 3 y. *D*, Scenario where individual was continuously enrolled starting at age 3 y and ending at age 12 y and does not have a claim for IMD. Abbreviations: CE, continuous enrollment; IMD, invasive meningococcal disease; y, years.

The baseline period (6 months prior to the index date) for the toddler and child cohorts may contain the follow-up period for the infant or toddler cohorts, respectively (Figure 1). As continuous enrollment prior to the index date was not a study requirement, length of the baseline period varied across toddlers and children which may have led to an underestimation of risk factors. Additionally, to capture IMD cases in the early months of life, no baseline period was considered for the infant cohorts. Individuals were followed until the first inpatient claim with an IMD diagnosis in the primary position, end of age for a given cohort, end of continuous enrollment (CE), death, or end of study period, whichever was earliest (variable follow-up).

### Variables

Demographic characteristics such as age, sex, region (commercially insured only), race/ethnicity (Medicaid-insured only), and birth hospitalization (infant cohorts only) were measured on the index date for all age cohorts. In the variable follow-up period for infants and in the 6 months prior to the index date for the toddler and child cohorts, clinical characteristics, such as meningococcal vaccination, conditions known or hypothesized to be associated with increased IMD risk (functional/anatomic asplenia, complement component deficiency, sickle cell anemia, asthma, autoimmune disease, immunocompromised status, hemophilia, liver disease, malignancy, preterm delivery, renal disease), and medications known or hypothesized to increase IMD risk (IMD-related medications; eculizumab, ravulizumab, corticosteroids) were documented.

### Outcomes

The following outcomes were evaluated in this study: number of individuals with IMD diagnosis, time at risk for IMD, IR, and incidence rate ratio (IRR). Invasive meningococcal disease incidence was characterized by patient-level demographics on the index date; evaluation by clinical characteristics occurred during the time at risk for the infant cohort and during the 6 months prior to index in the toddler and child cohorts. Invasive meningococcal disease incidence was characterized by month of age in the infant cohort (to better understand how IMD risk evolves during the first year of life) and by year of age in the toddler and child cohorts (to comprehensively evaluate age-related differences). Time at risk for IMD was calculated as number of days from index date to first IMD occurrence in patients with an IMD event; patients without an IMD event were censored at the end of their follow-up time. IRs were calculated as the number of patients with IMD per person-time at risk for IMD, reported per 100 000 person-years (PY). For all age cohorts, IRs were reported as overall IR and by demographic/clinical characteristics. Projected IRs (applied to the commercially insured cohorts only) provided estimates of IMD IRs nationally for all cohorts with ESI by patient demographic, clinical, and geographic characteristics. IRRs were calculated by comparing the IMD IR in all cohorts by patient demographic/clinical characteristics relative to a reference group in the same cohort.

### Analysis

Separate descriptive analyses were conducted for the commercially and Medicaid-insured populations and for each age cohort. A missing/unknown category was included for missing demographic data on sex, region, and urbanicity to understand the impact of missing demographic data.

To estimate IMD incidence in children aged <11 years with ESI, IRs were projected to the national population of children with ESI using MarketScan National Weights. Due to changes in the calculation of the national weights in 2008, projected IMD IRs from 2005 to 2007 and 2008 to 2022 were estimated separately.

For IRRs, 95% CIs were calculated using univariate Poisson regression models with an offset for PY.

### Patient Consent Statement

This study complied with all US patient confidentiality requirements, including the 1996 Health Insurance Portability and Accountability Act regulations. All data analyzed in this study were retrospective and de-identified; no direct subject contact or primary collection of individual human subject data occurred. Therefore, this study was considered non-human subjects research and did not require informed consent, ethics committee, or Institutional Review Board review per the 45 CFR 46 Category 4 Common Rule [23].

## RESULTS

### Population

A total of 29 103 698 commercially insured patients were identified, of whom 7 181 679 were included in the infant cohort, 13 628 626 in the toddler cohort, and 18 506 739 in the child cohort. A total of 17 906 057 Medicaid-insured patients were identified, of whom 7 417 412 were included in the infant cohort, 10 730 394 in the toddler cohort, and 9 588 361 in the child cohort.

### Invasive Meningococcal Disease Incidence Rate

#### Infant Cohort (Aged <1 Year)

**Figure 2.**
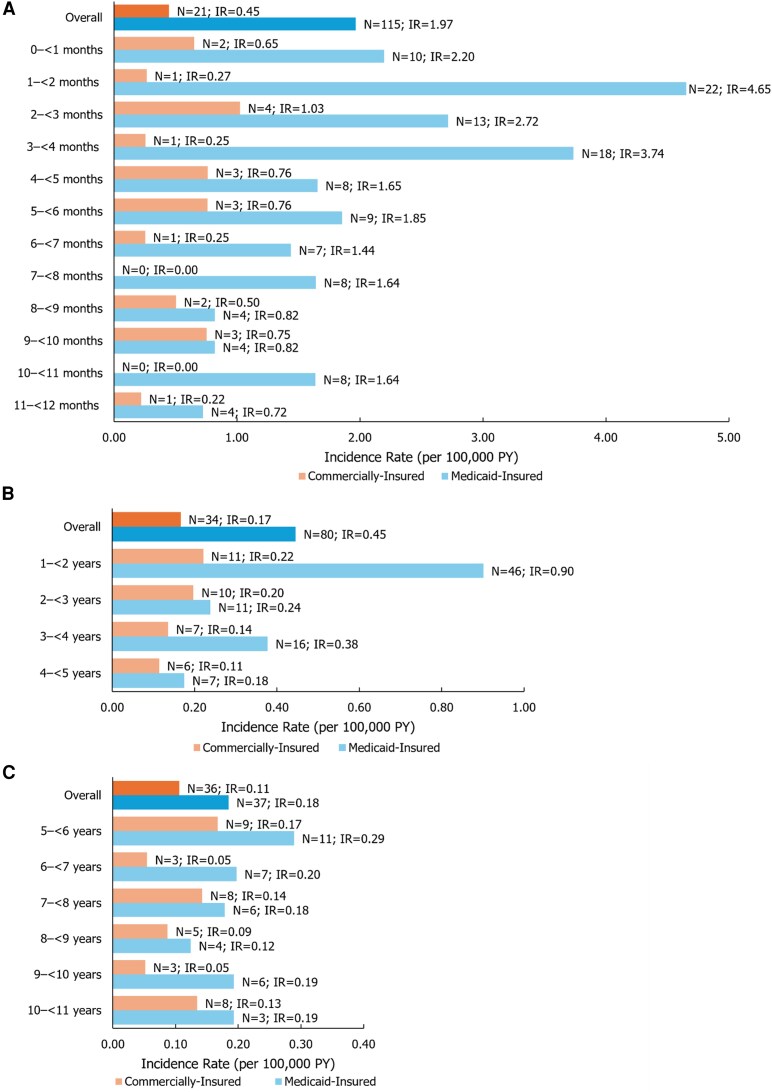
Incidence rate of IMD, 2005–2022 among the *A*, infant cohorts, *B*, toddler cohorts, and *C*, child cohorts. Abbreviations: IMD, invasive meningococcal disease; IR, incidence rate; PY, person-years.

Among commercially insured infants, there were 21 cases of IMD (IR: 0.45 per 100 000 PY); IRs varied by month of age, with the highest IR among infants aged 2 to <3 months (1.03 per 100 000 PY; Figure 2*A*). When the observed IRs for the commercially insured cohort were projected to the US population with ESI, the overall IMD IRs were 0.98 per 100 000 PY (2005–2007) and 0.29 per 100 000 PY (2008–2022). For the most recent years (2008–2022), projected IRs were highest for infants aged 2 to <3 months (1.14 per 100 000 PY; Table 1).

**Table 1.** Projected Incidence Rate of IMD by Demographic, Clinical, and Geographic Characteristics, Commercially insured Infants, 2005–2022

	Commercial: 2005–2007			Commercial: 2008–2022		
	N	IR^a^	IRR (95% CI)	N	IR^a^	IRR (95% CI)
**Demographic characteristics^b^**						
**Sex**						
Male (REF)	42	1.00	REF	21	0.28	REF
Female	38	0.95	0.96 (0.62, 1.49)	22	0.30	1.09 (0.60, 1.97)
**Age on index**						
0–3 months (REF)	79	1.21	REF	34	0.26	REF
4–6 months	0	-	-	3	0.33	1.28 (0.42, 3.88)
7–9 months	0	-	-	2	0.40	1.53 (0.41, 5.71)
10–12 months	0	-	-	3	1.56	6.00 (1.98, 18.2)
**Age on incidence^c^**						
Overall	79	0.98	…	44	0.29	…
0 to <1 months	9	2.06	…	3	0.30	…
1 to <2 months	21	3.79	…	0	-	…
2 to <3 months	0	-	…	15	1.14	…
3 to <4 months	0	-	…	3	0.25	…
4 to <5 months	7	1.12	…	5	0.41	…
5 to <6 months	22	3.31	…	6	0.43	…
6 to <7 months	0	-	…	3	0.27	…
7 to <8 months	0	-	…	0	-	…
8 to <9 months	11	1.45	…	3	0.23	…
9 to <10 months	0	-	…	6	0.47	…
10 to <11 months	0	-	…	0	-	…
11 to <12 months	9	1.00	…	0	-	…
**Index year**						
2005 (REF)	79	2.87	REF	…	…	…
2006	0	-	-	…	…	…
2007	0	-	-	…	…	…
2008	…	…	…	3	0.30	REF
2009	…	…	…	8	0.78	2.58 (0.69, 9.59)
2010	…	…	…	5	0.55	1.82 (0.44, 7.57)
2011	…	…	…	2	0.26	0.86 (0.16, 4.66)
2012	…	…	…	6	0.67	2.24 (0.56, 8.88)
2013	…	…	…	0	-	-
2014	…	…	…	6	0.75	2.49 (0.64, 9.68)
2015	…	…	…	3	0.29	0.97 (0.20, 4.65)
2016	…	…	…	3	0.29	0.96 (0.20, 4.62)
2017	…	…	…	7	0.64	2.11 (0.56, 8.01)
2018	…	…	…	0	-	-
2019	…	…	…	0	-	-
2020	…	…	…	0	-	-
2021	…	…	…	0	-	-
2022	…	…	…	0	-	-
**Medical condition/medication use potentially increasing IMD risk^d^**						
**Clinical conditions^e^**						
Asthma	7	2.13	2.31 (1.08, 4.93)	0	-	-
Autoimmune disease	19	3.03	3.78 (2.27, 6.31)	0	-	-
Complement component deficiency	0	-	-	3	391.15	1 457 (463, 4 583)
Preterm delivery	7	2.13	2.30 (1.08, 4.91)	3	0.39	1.37 (0.43, 4.35)
Renal disease	0	-	-	6	18.80	74.60 (30.9, 180)
**IMD-related medication use^e^**						
Corticosteroid	18	4.50	5.66 (3.35, 9.59)	3	0.43	1.52 (0.46, 5.01)
Oral	7	1.07	1.10 (0.52, 2.36)	0	-	-
Inhaled	7	2.64	2.88 (1.35, 6.15)	3	0.92	3.32 (1.01, 10.95)
Intravenous	18	16.87	22.00 (13.0, 37.3)	0	-	-
Other	0	-	-	0	-	-
Eculizumab	0	-	-	0	-	-
Ravulizumab	0	-	-	0	-	-
**IMD vaccination^e^**	0	-	-	0	-	-
**Number of medical conditions/medications^f^**						
0	53	0.77	REF	32	0.24	REF
1	19	1.68	2.19 (1.30, 3.69)	12	0.67	2.75 (1.41, 5.35)
2	7	7.43	9.70 (4.49, 20.97)	0	-	-
3+	0	-	-	0	-	-
**Geographic characteristics^b^**						
**Population density**						
Urban (REF)	60	0.84	REF	41	0.30	REF
Rural	19	1.97	2.36 (1.41, 3.94)	3	0.20	0.66 (0.20, 2.13)
Unknown	0	-	-	0	-	-
**Census geographic region^g^**						
Northeast	…	2.42	REF	…	0.18	REF
New England	…	-	-	…	-	-
Middle Atlantic	…	3.56	REF	…	0.25	REF
North Central	…	-	-	…	0.28	1.57 (0.53, 4.65)
East North Central	…	-	-	…	-	-
West North Central	…	-	-	…	0.84	3.38 (1.14, 10.0)
South	…	0.66	0.27 (0.15, 0.48)	…	0.32	1.79 (0.65, 4.97)
South Atlantic	…	-	-	…	0.42	1.68 (0.57, 4.92)
East South Central	…	2.64	0.74 (0.36, 1.52)	…	-	-
West South Central	…	0.85	0.24 (0.11, 0.52)	…	0.32	1.29 (0.37, 4.47)
West	…	1.00	0.41 (0.24, 0.70)	…	0.35	1.96 (0.69, 5.61)
Mountain	…	1.68	0.47 (0.24, 0.92)	…	0.25	1.02 (0.24, 4.31)
Pacific	…	0.67	0.19 (0.09, 0.39)	…	0.39	1.59 (0.53, 4.75)
**HHS region^g^**						
Region 1	…	-	-	…	-	-
Region 2	…	5.74	REF	…	0.36	REF
Region 3	…	-	-	…	0.42	1.18 (0.34, 4.06)
Region 4	…	0.81	0.14 (0.07, 0.29)	…	0.20	0.56 (0.17, 1.90)
Region 5	…	-	-	…	-	-
Region 6	…	0.77	0.13 (0.06, 0.28)	…	0.31	0.85 (0.25, 2.94)
Region 7	…	-	-	…	1.16	3.24 (1.09, 9.59)
Region 8	…	3.37	0.59 (0.30, 1.16)	…	-	-
Region 9	…	0.70	0.12 (0.06, 0.25)	…	0.55	1.54 (0.54, 4.40)
Region 10	…	-	-	…	-	-

Abbreviations: CI, confidence interval; HHS, Health and Human Services; HIV, human immunodeficiency virus; IMD: invasive meningococcal disease; IR, incidence rate; IRR, incidence rate ratio; PY, person-years; REF, reference.

^a^IR reported per 100 000 PY.

^b^Measured on index date.

^c^Person-time at risk was calculated during each age interval; individuals could contribute person-time to multiple age ranges based on length of continuous enrollment.

^d^Measured during the variable follow-up period.

^e^For each medical condition/medication use the referent group is individuals without each medical condition/medication use.

^f^Medical conditions/medications include: asplenia, asthma, autoimmune disease, complement component deficiency, hemophilia, HIV infection, hematopoietic stem cell transplant, immunodeficiency, organ transplant, liver disease, malignancy, preterm delivery, renal disease, sickle cell anemia, eculizumab, and ravulizumab.

^g^Number of cases cannot be reported in commercial for regions with <30 cases.

Compared with the commercially insured cohort, IMD IRs were higher overall and for each month of age in Medicaid-insured infants. There were 115 cases of IMD overall (IR: 1.97 per 100 000 PY), with IRs varying by month of age from 0.72 to 4.65 per 100 000 PY. IRs were higher in infants aged <4 months than those ≥4 months (highest IR of 4.65 per 100 000 PY at 1 to <2 months; Figure 2*A*).

#### Toddler Cohort (Aged 1–4 Years)

There were 34 cases of IMD (IR: 0.17 per 100 000 PY) in the commercially insured toddler cohort. IRs decreased as age increased, from 0.22 to 0.11 per 100 000 PY for toddlers aged 1 to <2 and 4 to <5 years, respectively (Figure 2*B*). Projected IMD IRs were 0.38 (2005–2007) and 0.10 (2008–2022) per 100 000 PY (Supplementary Table 1).

Among Medicaid-insured toddlers, 80 IMD cases occurred, resulting in an IR of 0.45 per 100 000 PY (Figure 2*B*). Toddlers aged 1 to <2 years had the highest IR and those aged 4 to <5 years had the lowest IR (0.90 and 0.18 per 100 000 PY, respectively).

#### Child Cohort (Aged 5–10 Years)

In the commercially insured child cohort, there were 36 cases of IMD (IR: 0.11 per 100 000 PY; Figure 2*C*); the IR projected to the commercially insured US population was higher in the years 2005–2007 (0.19 per 100 000 PY), but decreased to 0.05 per 100 000 PY in the period from 2008 to 2022 (Supplementary Table 2).

There were 37 IMD cases (IR: 0.18 per 100 000 PY) in the Medicaid-insured child cohort (Figure 2*C*). No pattern in IRs based on age of diagnosis in the child cohort was observed in either dataset.

#### Incidence Rate of Invasive Meningococcal Disease by Demographic, Clinical, and Geographic Characteristics

The following results are calculated from low IMD case counts, reflecting that IMD is an uncommon disease in the United States. Further investigation is warranted to fully evaluate these associations.

#### Infant Cohort (Aged <1 Year)

Of the commercially insured infants with IMD, 1 had complement component deficiency (IR: 303.44 per 100 000 PY) and 2 had renal disease (IR: 27.06 per 100 000 PY), resulting in higher IRs compared with infants without those conditions (IRR [95% CI]: 714.14 [95.84, 5 321.38] and 67.07 [15.62, 287.95], respectively; Table 2).

**Table 2.** Incidence Rate of IMD by Demographic, Clinical, and Geographic Characteristics, Infant Cohorts, 2005–2022

	Commercial			Medicaid		
	N = 7 181 679			N = 7 417 412		
	N	IR^a^	IRR (95% CI)	N	IR^a^	IRR (95% CI)
**Demographic characteristics^b^**						
**Sex**						
Male (REF)	11	0.45	REF	69	2.31	REF
Female	10	0.44	0.96 (0.40, 2.28)	46	1.61	0.70 (0.49, 0.99)
**Race^c^**						
White (REF)	…	…	…	75	3.11	REF
Black	…	…	…	13	0.82	0.26 (0.15, 0.46)
American Indian	…	…	…	1	2.95	0.95 (0.14, 6.25)
Hispanic	…	…	…	9	1.45	0.47 (0.24, 0.90)
Asian or Pacific Islands	…	…	…	2	2.31	0.74 (0.19, 2.84)
Other	…	…	…	15	1.34	0.31 (0.05, 2.01)
**Age on index**						
0–3 months (REF)	18	0.44	REF	107	1.98	REF
4–6 months	1	0.28	0.63 (0.08, 4.69)	4	1.57	0.79 (0.29, 2.15)
7–9 months	1	0.48	1.07 (0.14, 8.05)	4	3.02	1.52 (0.56, 4.12)
10–12 months	1	1.28	2.90 (0.39, 21.73)	0	-	-
**Index year**						
2005 (REF)	6	2.41	REF	25	3.73	REF
2006	0	-	-	10	4.17	1.12 (0.54, 2.33)
2007	0	-	-	11	4.50	1.21 (0.59, 2.46)
2008	1	0.26	0.11 (0.01, 0.89)	12	3.22	0.87 (0.44, 1.72)
2009	3	0.81	0.34 (0.08, 1.35)	10	2.86	0.77 (0.37, 1.60)
2010	2	0.57	0.24 (0.05, 1.18)	7	2.17	0.58 (0.25, 1.35)
2011	2	0.52	0.22 (0.04, 1.07)	3	1.04	0.28 (0.08, 0.92)
2012	2	0.58	0.24 (0.05, 1.20)	6	1.56	0.42 (0.17, 1.02)
2013	0	-	-	2	0.57	0.15 (0.04, 0.65)
2014	2	0.72	0.30 (0.06, 1.47)	2	0.58	0.16 (0.04, 0.66)
2015	1	0.37	0.15 (0.02, 1.28)	6	1.63	0.44 (0.18, 1.07)
2016	1	0.41	0.17 (0.02, 1.41)	2	0.59	0.16 (0.04, 0.67)
2017	1	0.44	0.19 (0.02, 1.54)	7	2.24	0.60 (0.26, 1.39)
2018	0	-	-	2	0.67	0.18 (0.04, 0.76)
2019	0	-	-	3	0.93	0.25 (0.08, 0.83)
2020	0	-	-	5	1.69	0.46 (0.17, 1.19)
2021	0	-	-	2	0.86	0.23 (0.05, 0.98)
2022	0	-	-	0	-	-
**Medical condition/medication use potentially increasing IMD risk^d^**						
**Clinical conditions^e^**						
Asthma	1	0.69	1.58 (0.21, 11.76)	5	1.47	0.74 (0.30, 1.81)
Autoimmune disease	2	0.61	1.41 (0.33, 6.05)	16	2.97	1.60 (0.94, 2.71)
Complement component deficiency^f^	1	303.44	714.14 (95.84, 5321.38)	1	269.62	138.06 (19.28, 988.59)
Hemophilia	0	-	-	1	31.23	16.01 (2.24, 114.63)
Immunocompromised	0	-	-	1	26.94	13.82 (1.93, 98.92)
Liver disease	0	-	-	1	3.06	1.56 (0.22, 11.17)
Preterm delivery	2	0.83	1.95 (0.46, 8.39)	19	3.55	1.96 (1.20, 3.21)
Renal disease	2	27.06	67.07 (15.62, 287.95)	3	20.53	10.70 (3.40, 33.66)
**IMD-related medication use^e^**						
Corticosteroid	3	1.33	3.32 (0.98, 11.26)	8	2.16	1.10 (0.54, 2.26)
Oral	1	0.26	0.56 (0.08, 4.17)	15	2.10	1.08 (0.63, 1.85)
Inhaled	2	1.62	3.91 (0.91, 16.78)	3	1.34	0.67 (0.21, 2.12)
Intravenous	2	2.07	5.04 (1.17, 21.62)	4	2.80	1.44 (0.53, 3.90)
Other	0	-	-	1	2.82	1.44 (0.20, 10.31)
Eculizumab	0	-	-	0	-	-
Ravulizumab	0	-	-	0	-	-
**IMD vaccination^e^**	0	-	-	1	25.26	12.96 (1.81, 92.77)
**Number of medical conditions/medications^g^**						
0	14	0.35	REF	75	1.66	REF
1	6	0.96	2.76 (1.06, 7.18)	33	2.86	1.72 (1.15, 2.60)
2	1	1.87	5.37 (0.71, 40.9)	7	4.52	2.73 (1.26, 5.92)
3+	0	-	-	0	-	-
**Geographic characteristics^b^**						
**Population density**						
Urban	17	0.42	REF	81	1.86	REF
Rural	3	0.58	1.39 (0.41, 4.73)	33	2.48	1.33 (0.89, 2.00)
Unknown	1	1.20	2.90 (0.39, 21.79)	1	0.62	0.33 (0.05, 2.40)
**Census geographic region^h^**						
Northeast	…	0.53	REF	…	…	…
New England	…	-	-	…	…	…
Middle Atlantic	…	0.70	REF	…	…	…
North Central	…	0.18	0.35 (0.06, 1.91)	…	…	…
East North Central	…	-	-	…	…	…
West North Central	…	0.64	0.91 (0.17, 4.99)	…	…	…
South	…	0.43	0.82 (0.25, 2.73)	…	…	…
South Atlantic	…	0.42	0.61 (0.15, 2.44)	…	…	…
East South Central	…	0.38	0.55 (0.06, 4.88)	…	…	…
West South Central	…	0.47	0.67 (0.15, 3.01)	…	…	…
West	…	0.65	1.23 (0.35, 4.36)	…	…	…
Mountain	…	0.61	0.87 (0.16, 4.75)	…	…	…
Pacific	…	0.67	0.97 (0.24, 3.88)	…	…	…
Unknown	…	1.17	2.21 (0.25, 19.76)	…	…	…
**HHS region^h^**						
Region 1	…	-	-	…	…	…
Region 2	…	1.04	REF	…	…	…
Region 3	…	0.44	0.42 (0.08, 2.29)	…	…	…
Region 4	…	0.32	0.31 (0.07, 1.36)	…	…	…
Region 5	…	-	-	…	…	…
Region 6	…	0.45	0.43 (0.1, 1.92)	…	…	…
Region 7	…	0.85	0.81 (0.15, 4.44)	…	…	…
Region 8	…	0.63	0.61 (0.07, 5.42)	…	…	…
Region 9	…	0.87	0.83 (0.22, 3.08)	…	…	…
Region 10	…	-	-	…	…	…
Unknown	…	1.17	1.12 (0.12, 9.98)	…	…	…

Abbreviations: CI, confidence interval; HHS, Health and Human Services; HIV, human immunodeficiency virus; IMD, invasive meningococcal disease; IR, incidence rate; IRR, incidence rate ratio; PY, person-years; REF, reference.

^a^IR reported per 100 000 PY.

^b^Measured on index date.

^c^Race is not available for the commercial cohort.

^d^Measured during the variable length follow-up period.

^e^For each condition/medication the referent group is individuals without each medical condition/medication use.

^f^In both the Commercial and Medicaid cohort 1 infant with IMD had a diagnosis of complement component deficiency prior to the diagnosis of IMD.

^g^Medical conditions/medications include: asplenia, asthma, autoimmune disease, complement component deficiency, hemophilia, HIV infection, hematopoietic stem cell transplant, immunodeficiency, organ transplant, liver disease, malignancy, preterm delivery, renal disease, sickle cell anemia, eculizumab, and ravulizumab.

^h^Region is not reportable for the Medicaid cohort due to data use agreements with MarketScan data contributors; number of cases cannot be reported in commercial for regions with <30 cases.

Projected IRs from 2005 to 2007 were higher for commercially insured infants with asthma (IRR [95% CI]: 2.31 [1.08, 4.93]), autoimmune disease (IRR [95% CI]: 3.78 [2.27, 6.31]), and preterm delivery (IRR [95% CI]: 2.30 [1.08, 4.91]) compared with infants without these comorbidities (Table 1). Similarly, projected IRs from 2008 to 2022 for infants with complement component deficiency and renal disease were higher than for infants without those conditions (IRR [95% CI]: 1 457 [463, 4 583] and 74.60 [30.9, 180], respectively). In the years from 2005 to 2007, infants who lived in rural counties versus urban counties had a higher projected IMD IR (IRR [95% CI]: 2.36 [1.41, 3.94]).

In the Medicaid-insured cohort, 1 infant with IMD had complement component deficiency (IR: 269.62 per 100 000 PY; IRR [95% CI]: 138.06 [19.28, 988.59]; Table 2). Incidence rates were also higher for infants who were immunocompromised (IRR [95% CI]: 13.82 [1.93, 98.92]), had hemophilia (IRR [95% CI]: 16.01 [2.24, 114.63]), and had renal disease (IRR [95% CI]: 10.70 [3.40, 33.66]), compared with infants without these comorbidities. Furthermore, IMD IR was higher in infants born prematurely compared with those without a preterm delivery (IRR [95% CI]: 1.96 [1.20, 3.21]).

#### Toddler Cohort (Aged 1–4 Years)

Among commercially insured toddlers, 2 had asthma (IR: 0.70 per 100 000 PY), resulting in a higher IR compared with toddlers without IMD (IRR [95% CI]: 4.43 [1.06, 18.47]; Supplementary Table 3). There were no observed differences in IRs for any clinical condition or IMD-related medication use in the Medicaid-insured toddler cohort.

Commercially insured female toddlers had a lower projected IMD IR than male toddlers during both time periods (2005–2007 IRR [95% CI]: 0.44 [0.33, 0.59]; 2008–2022 IRR [95% CI]: 0.48 [0.35, 0.66]; Supplementary Table 1). In 2008–2022, commercially insured toddlers with asthma had a higher projected IMD IR than those without asthma (IRR [95% CI]: 18.60 [12.8, 26.9]). The projected IMD IR was higher among commercially insured toddlers in rural counties than those in urban counties in both time periods (2005–2007 IRR [95% CI]: 1.53 [1.09, 2.15]; 2008–2022 IRR [95% CI]: 2.66 [1.89, 3.74]).

#### Child Cohort (Aged 5–10 Years)

In the commercially insured child cohort, IMD IRs were higher in children aged 7 to <8 years and aged 10 to <11 years relative to those aged 5 to <6 years on index (IRR [95% CI]: 2.59 [1.08, 6.17] and 3.96 [1.13, 13.76], respectively; Supplementary Table 4). Children who were immunocompromised were at greater risk of IMD than children who were not (IRR [95% CI]: 133.08 [18.23, 971.40]).

In line with the infant and toddler cohorts, the projected IR in 2005–2007 was higher in children who lived in rural versus urban counties (IRR [95% CI]: 6.72 [95% CI: 5.48, 8.24]; Supplementary Table 2). Female children had a higher projected IMD IR than male children in both time periods (2005–2007 IRR [95% CI]: 1.69 [1.37, 2.09]; 2008–2022 IRR [95% CI]: 1.27 [1.01, 1.60]). The highest projected IRR by age was observed among commercially insured children aged 10 to <11 years versus those aged 5 to <6 years on index in 2005–2007 (IRR [95% CI]: 7.98 [5.37, 11.85]).

In the Medicaid-insured cohort, IMD IRs were higher in children aged 6 to <7 years (IRR [95% CI]: 3.43 [1.46, 8.01]) and aged 8 to <9 years (IRR [95% CI]: 3.66 [1.43, 9.36]) compared with children aged 5 to <6 years on index (Supplementary Table 4). Additionally, IMD IR was higher in children with versus without sickle cell anemia (IRR [95% CI]: 26.56 [3.64, 193.69]). No other differences in IMD IRs were observed in the Medicaid population.

## DISCUSSION

While IMD is uncommon, the risks and consequences associated with the disease are substantial. Overall IMD incidence was low, though incidence varied by insurance type and certain demographic and clinical characteristics. Invasive meningococcal disease IRs were highest in infants, particularly those aged <4 months, and were generally higher among Medicaid- versus commercially insured individuals. Certain clinical conditions, such as immunocompromised status, were also commonly associated with higher IMD IRs. These findings provide further evidence of the burden of IMD in individuals aged <11 years. Overall incidences of IMD reported among infants and toddlers in the current study are comparable to annual CDC reports, while the incidence in children was higher than in CDC reports [10, 13–16]. Notably, the latest CDC national data on IMD incidence are reported by overall age ranges and not stratified by insurance coverage [10, 13–16], while our findings stratify incidence by month (infants) and year (toddlers and children) and by insurance type, highlighting substantial disparities corresponding to age and insurance coverage that may be masked in overall population statistics. Many clinical conditions associated with increased IMD incidence were observed only in the Medicaid-insured cohort, and IMD IRs overall were higher in Medicaid- versus commercially insured individuals across all 3 age cohorts. The observed disparities between commercially and Medicaid-insured cohorts may reflect differences in socioeconomic status, healthcare access, and geographic distribution. Future studies should explore these factors to inform targeted interventions.

Complement component deficiency has been well established to increase IMD risk in child and adult populations [24–26]. To our knowledge, this is the first report extending the association of complement component deficiency with an increase in IMD incidence in infants. Furthermore, preterm delivery, immunodeficiency, renal disease, and hemophilia were associated with increased IMD incidence in the Medicaid-insured infant cohort, with the latter 2 conditions not previously documented for their association with IMD risk in infants. However, noting the low IMD case counts in this study, further research on the associations between IMD and certain clinical characteristics (eg, hemophilia and renal disease) is required to clearly validate the associations described here.

In alignment with previous findings showing the association of chronic respiratory conditions with increased IMD risk [26], we observed asthma was associated with increased IMD risk among commercially insured individuals in the present study. Prematurity and low birth weight have likewise been previously associated with a higher risk of IMD, with some studies showing effects of prematurity may persist into adolescence and young adulthood [27–29]. In this study, across the commercially and Medicaid-insured cohorts, IMD incidence was approximately 2 times higher in infants born prematurely than in those carried to full term, though this association was not statistically significant among commercially insured infants. Additional research to further elucidate the long-term impact of prematurity and low birth weight on IMD risk may be warranted [27–30].

Evaluation of IMD incidence among pediatric populations is crucial for understanding the overall burden of the disease. The consequences of IMD can be substantial for both patients and their caregivers, and extend beyond the acute phase with severe long-term sequelae and negative impacts on physical, social, emotional, functional, and economic quality of life [31–34]. Despite IMD incidence being highest in infants and young children, most existing studies focus on older populations such as young adults, creating a gap in the literature that may underestimate the above impacts. Highlighting the burden of disease in infants and young children is particularly important as the majority of these individuals are not currently eligible for protection with vaccines. Greater awareness of the impact of IMD on pediatric populations provides important context for families, healthcare providers, and policy decision-makers with regard to improving IMD-related health outcomes [35–40]. Targeted mitigation strategies may reduce IMD burden among high-risk groups, but the difficulty of identifying individuals at an early age for increased risk may necessitate a broader approach.

### Study Limitations

Invasive meningococcal disease is an uncommon disease, and the number of cases identified was expected to be small in each age cohort, limiting the stratification of results and requiring further investigation. This study was descriptive in nature and the low case numbers reported here are not a weakness of the databases, but a reflection of the low IMD incidence in the United States. The broad study period aimed to capture a more complete picture of IMD incidence. Increased risk of IMD in some conditions and medications known or hypothesized to be associated with increased IMD risk may not have been observed in this study due to no or few cases captured within study parameters. The lack of observed associations may be attributable to the rarity of IMD, as well as limited population size and/or follow-up time among those with high-risk conditions and medications.

A validated algorithm to identify IMD in administrative claims databases, where laboratory confirmation of IMD was not available and cases were inferred from the presence of diagnosis codes on non-rule-out/diagnostic medical claims, was not available. The study may underestimate IMD incidences as the IMD diagnoses included were limited to those from an inpatient setting in the primary position, consistent with recent research on IMD incidence using administrative claims databases [20, 22]. Patients with an IMD diagnosis in the emergency department or outpatient setting who died prior to inpatient admission were not captured in this study. Additionally, serogroup-specific data were not available in the databases to confirm serogroup-specific trends in this study.

Many of the clinical characteristics shown or hypothesized to increase risk for IMD were anticipated to have very low prevalence in infants, toddlers, and children. The expected low prevalence of these clinical characteristics combined with the low IMD incidence in the United States may lead to unreliable incidence estimations in some subgroups of individuals. Nonetheless, to our knowledge, this is the first study to examine conditions shown or hypothesized to increase IMD risk extended to infants, toddlers, and children.

Using administrative healthcare databases requires relying solely on claims submitted by providers for reimbursement as a surrogate for clinical details rather than medical records with confirmed diagnoses. Additionally, the MarketScan Commercial and Multi-State Medicaid Databases do not capture the entire US population. As this study was limited to infants, toddlers, and children with commercial or Medicaid insurance, the results of this analysis may not be generalizable, including to those with other insurance or without health insurance. However, more than 90% of infants, toddlers, and children have health insurance in the United States [41], so results may be generalizable to a substantial portion of children aged <11 years in the United States.

### Conclusions

Overall, incidence of IMD was low among children aged <11 years in the United States, but differed across age, insurance type, and demographic and clinical characteristics. IRs were highest among Medicaid-insured infants, infants aged <4 months, and infants with certain clinical conditions; earlier preventative interventions may reduce the incidence and impacts of IMD in early life.

## Supplementary Material

ofag185_Supplementary_Data

## Supplementary Data

Supplementary materials are available at *Open Forum Infectious Diseases* online. Consisting of data provided by the authors to benefit the reader, the posted materials are not copyedited and are the sole responsibility of the authors, so questions or comments should be addressed to the corresponding author.
