# Correction to: Risk of Invasive Meningococcal Disease in Children Aged <11 Years in the United States

**DOI:** 10.1093/ofid/ofag375

**Published:** 2026-06-22

This is a correction to: Oscar Herrera-Restrepo, Elizabeth Packnett, Megan K Richards, Elise Kuylen, Tosin O Olaiya, Thatiana Pinto, Lindsay C Landgrave, Ryan Ross, Andrew G Allmon, Risk of Invasive Meningococcal Disease in Children Aged <11 Years in the United States, *Open Forum Infectious Diseases*, Volume 13, Issue 4, April 2026, ofag185, https://doi.org/10.1093/ofid/ofag185

The following changes have been made to the originally published paper.

The date range for the real-world data in the graphical abstract has been amended to begin at “01/01/2005” instead of “01/01/2025”.

