# Supplementary material for: Risk of Invasive Meningococcal Disease in Children Aged <11 Years in the United States
Source: Open Forum Infect Dis. 2026 Apr 10;13(4):ofag185. doi: 10.1093/ofid/ofag185 (PMC13095375; doi:10.1093/ofid/ofag185)
Supplement: ofag185_Supplementary_Data [file ofag185_supplementary_data.docx]

**Risk of Invasive Meningococcal Disease in Children Aged <11 Years in the United States**

Oscar Herrera-Restrepo (ORCID: 0000-0001-8193-999X)^1^, Elizabeth Packnett (ORCID: 0000-0002-4731-6956)^2^, Megan K. Richards (ORCID:0000-0003-0737-389X)^2^, Elise Kuylen (ORCID: 0000-0002-3528-5507)^3^, Tosin O. Olaiya (ORCID: 0000-0002-5049-0111)^1^, Thatiana Pinto (ORCID: 0000-0002-0957-3118)^3^, Lindsay C. Landgrave (ORCID: 0000-0001-8954-9064)^1^, Ryan Ross (ORCID:0000-0002-9316-5810)^2^, Andrew G. Allmon (ORCID:0009-0004-6879-9231)^4^

^1^GSK, Philadelphia, PA, USA

^2^Merative, Ann Arbor, MI, USA

^3^GSK, Wavre, Belgium

^4^GSK, Durham, NC, USA

**Correspondence to:** Tosin Olaiya

Email: tosin.o.olaiya@gsk.com

Tosin Olaiya, MBChB, MSc

Director, Medical Affairs, Vaccines

US Medical Affairs

GSK

FMC Tower Suite 1700

2929 Walnut Street, Philadelphia, PA, USA 19104

**Alternate Corresponding Author:** Thatiana Pinto

Email: thatiana.x.pinto@gsk.com

Thatiana Pinto, PhD

GSK

Avenue Fleming, 20, 1300 Wavre, Belgium

**Journal:** *Open Forum Infectious Diseases*

**SUPPLEMENTARY APPENDIX**

**SUPPLEMENTARY MATERIALS**

Supplemental Table 1. Projected incidence rate by demographic, clinical, and geographic characteristics, commercially-insured toddler cohort, 2005–2022

|  | **Commercial: 2005–2007** | | | **Commercial: 2008–2022** | | |
| --- | --- | --- | --- | --- | --- | --- |
|  | N | IR^a^ | IRR (95% CI) | N | IR^a^ | IRR (95% CI) |
| **Demographic Characteristics^b^** | | | | | | |
| **Sex** |  |  |  |  |  |  |
| Male (REF) | 159 | 0.52 | REF | 125 | 0.14 | REF |
| Female | 66 | 0.23 | 0.44 (0.33, 0.59) | 56 | 0.07 | 0.48 (0.35, 0.66) |
| **Age on index** |  |  |  |  |  |  |
| 1–<2 years (REF) | 166 | 0.44 | REF | 141 | 0.12 | REF |
| 2–<3 years | 31 | 0.28 | 0.63 (0.43, 0.93) | 20 | 0.08 | 0.67 (0.42, 1.07) |
| 3–<4 years | 28 | 0.35 | 0.81 (0.54, 1.21) | 8 | 0.04 | 0.38 (0.19, 0.76) |
| 4–<5 years | 0 | - | - | 11 | 0.14 | 1.25 (0.68, 2.29) |
| **Age on incidence^c^** |  |  |  |  |  |  |
| Overall | 225 | 0.38 |  | 181 | 0.10 |  |
| 1–<2 years | 42 | 0.36 |  | 91 | 0.20 |  |
| 2–<3 years | 112 | 0.78 |  | 17 | 0.04 |  |
| 3–<4 years | 28 | 0.17 |  | 61 | 0.14 |  |
| 4–<5 years | 44 | 0.25 |  | 11 | 0.03 |  |
| **Index year** |  |  |  |  |  |  |
| 2005 (REF) | 116 | 0.56 | REF |  |  |  |
| 2006 | 43 | 0.22 | 0.39 (0.28, 0.55) |  |  |  |
| 2007 | 66 | 0.34 | 0.61 (0.45, 0.83) |  |  |  |
| 2008 |  |  |  | 35 | 0.28 | REF |
| 2009 |  |  |  | 38 | 0.27 | 0.97 (0.61, 1.53) |
| 2010 |  |  |  | 5 | 0.05 | 0.19 (0.08, 0.48) |
| 2011 |  |  |  | 18 | 0.18 | 0.65 (0.37, 1.15) |
| 2012 |  |  |  | 0 | - | - |
| 2013 |  |  |  | 16 | 0.13 | 0.47 (0.26, 0.85) |
| 2014 |  |  |  | 18 | 0.18 | 0.64 (0.36, 1.13) |
| 2015 |  |  |  | 16 | 0.12 | 0.43 (0.24, 0.77) |
| 2016 |  |  |  | 9 | 0.07 | 0.24 (0.12, 0.50) |
| 2017 |  |  |  | 0 | - | - |
| 2018 |  |  |  | 0 | - | - |
| 2019 |  |  |  | 26 | 0.18 | 0.62 (0.37, 1.03) |
| 2020 |  |  |  | 0 | - | - |
| 2021 |  |  |  | 0 | - | - |
| 2022 |  |  |  | 0 | - | - |
| **Condition/Medication Use Potentially Increasing IMD Risk^d^** | | | | | | |
| **Clinical conditions^e^** |  |  |  |  |  |  |
| Asthma | 0 | - | - | 34 | 1.58 | 18.60 (12.8, 26.9) |
| Autoimmune disease | 0 | - | - | 8 | 0.26 | 2.62 (1.30, 5.26) |
| **IMD-related medications^e^** |  |  |  |  |  |  |
| Corticosteroid use | 0 | - | - | 5 | 0.13 | 1.29 (0.51, 3.25) |
| Oral | 0 | - | - | 42 | 0.72 | 8.81 (6.25, 12.43) |
| Inhaled | 0 | - | - | 5 | 0.26 | 2.55 (1.01, 6.42) |
| Intravenous | 0 | - | - | 0 | - | - |
| Eculizumab | 0 | - | - | 0 | - | - |
| Ravulizumab | 0 | - | - | 0 | - | - |
| **Number of medical conditions/medications^f^** |  |  |  |  |  |  |
| 0 (REF) | 225 | 0.39 | REF | 138 | 0.08 | REF |
| 1 | 0 | - | - | 42 | 0.71 | 8.62 (6.11, 12.16) |
| **Geographic Characteristics^b^** | | | | | | |
| **Population density** |  |  |  |  |  |  |
| Urban (REF) | 185 | 0.35 | REF | 137 | 0.09 | REF |
| Rural | 41 | 0.54 | 1.53 (1.09, 2.15) | 44 | 0.23 | 2.66 (1.89, 3.74) |
| Unknown | 0 | - | - | 0 | - | - |
| **Census geographic region^g^** |  |  |  |  |  |  |
| Northeast (REF) |  | 0.41 | REF |  | 0.11 | REF |
| New England |  | 0.76 | 3.18 (1.86, 5.44) |  | 0.33 | 13.20 (5.31, 32.67) |
| Middle Atlantic (REF) |  | 0.24 | REF |  | 0.03 | REF |
| North Central |  | 0.56 | 1.38 (0.98, 1.94) |  | 0.12 | 1.07 (0.70, 1.64) |
| East North Central |  | 0.43 | 1.81 (1.08, 3.04) |  | 0.09 | 3.60 (1.44, 9.01) |
| West North Central |  | 0.77 | 3.20 (1.92, 5.34) |  | 0.19 | 7.44 (2.97, 18.66) |
| South |  | 0.18 | 0.44 (0.28, 0.68) |  | 0.09 | 0.82 (0.54, 1.25) |
| South Atlantic |  | 0.37 | 1.52 (0.88, 2.62) |  | 0.02 | 0.60 (0.17, 2.06) |
| East South Central |  | - | - |  | 0.32 | 12.80 (5.19, 31.55) |
| West South Central |  | - | - |  | 0.10 | 3.90 (1.50, 10.13) |
| West |  | 0.40 | 0.99 (0.68, 1.43) |  | 0.09 | 0.82 (0.52, 1.29) |
| Mountain |  | - | - |  | 0.11 | 4.30 (1.61, 11.46) |
| Pacific |  | 0.59 | 2.46 (1.51, 4.01) |  | 0.09 | 3.42 (1.36, 8.60) |
| **HHS region^g^** |  |  |  |  |  |  |
| Region 1 (REF) |  | 0.76 | REF |  | 0.33 | REF |
| Region 2 |  | - | - |  | 0.04 | 0.11 (0.04, 0.27) |
| Region 3 |  | 0.58 | 0.76 (0.48, 1.20) |  | 0.03 | 0.10 (0.04, 0.27) |
| Region 4 |  | 0.17 | 0.22 (0.12, 0.42) |  | 0.10 | 0.29 (0.18, 0.48) |
| Region 5 |  | 0.40 | 0.53 (0.33, 0.84) |  | 0.08 | 0.25 (0.15, 0.42) |
| Region 6 |  | - | - |  | 0.14 | 0.42 (0.25, 0.71) |
| Region 7 |  | 0.95 | 1.25 (0.79, 1.97) |  | 0.26 | 0.78 (0.46, 1.32) |
| Region 8 |  | - | - |  | 0.10 | 0.31 (0.13, 0.74) |
| Region 9 |  | 0.47 | 0.61 (0.39, 0.96) |  | 0.09 | 0.28 (0.16, 0.48) |
| Region 10 |  | 0.61 | 0.80 (0.42, 1.51) |  | - | - |

a IR reported per 100,000 PY

b Measured on index date

c Person-time at risk was calculated during each age interval; individuals could contribute person-time to multiple age ranges based on length of continuous enrollment

d Measured during the 6-month baseline period

e For each medical condition/medication use the referent group is individuals without each medical condition/medication use

f Medical conditions/medications include: asplenia, asthma, autoimmune disease, complement component deficiency, hemophilia, HIV infection, hematopoietic stem cell transplant, immunodeficiency, organ transplant, liver disease, malignancy, preterm delivery, renal disease, sickle cell anemia, eculizumab, and ravulizumab

g Number of cases cannot be reported in commercial for regions with <30 cases

Abbreviations: CI: confidence interval; HHS: Health and Human Services; IMD: invasive meningococcal disease; IR: incidence rate; IRR: incidence rate ratio; PY: person-years; REF: reference

Supplemental Table 2. Projected incidence rate by demographic, clinical, and geographic characteristics, commercially-insured child cohort, 2005–2022

|  | | **Commercial: 2005–2007** | | | | | | **Commercial: 2008–2022** | | | | | |
| --- | --- | --- | --- | --- | --- | --- | --- | --- | --- | --- | --- | --- | --- |
|  | | N | | IR^a^ | | IRR (95% CI) | | N | | IR^a^ | | IRR (95% CI) | |
| **Demographic Characteristics^b^** | | | | | | | | | | | | | |
| **Sex** | |  | |  | |  | |  | |  | |  | |
| Male (REF) | | 141 | | 0.14 | | REF | | 128 | | 0.05 | | REF | |
| Female | | 227 | | 0.24 | | 1.69 (1.37, 2.09) | | 155 | | 0.06 | | 1.27 (1.01, 1.6) | |
| **Age on index** | |  | |  | |  | |  | |  | |  | |
| 5–<6 years (REF) | | 75 | | 0.08 | | REF | | 139 | | 0.04 | | REF | |
| 6–<7 years | | 79 | | 0.26 | | 3.38 (2.46, 4.64) | | 16 | | 0.04 | | 0.90 (0.54, 1.5) | |
| 7–<8 years | | 107 | | 0.40 | | 5.18 (3.86, 6.96) | | 44 | | 0.11 | | 2.65 (1.89, 3.72) | |
| 8–<9 years | | 0 | | - | | - | | 11 | | 0.03 | | 0.80 (0.44, 1.46) | |
| 9–<10 years | | 71 | | 0.47 | | 6.06 (4.38, 8.38) | | 25 | | 0.10 | | 2.36 (1.54, 3.61) | |
| 10–<11 years | | 36 | | 0.62 | | 7.98 (5.37, 11.85) | | 47 | | 0.07 | | 1.70 (1.22, 2.36) | |
| **Age on incidence^c^** | |  | |  | |  | |  | |  | |  | |
| Overall | | 368 | | 0.11 | |  | | 283 | | 0.05 | |  | |
| 5–<6 years | | 47 | | 0.17 | |  | | 106 | | 0.10 | |  | |
| 6–<7 years | | 14 | | 0.05 | |  | | 17 | | 0.02 | |  | |
| 7–<8 years | | 92 | | 0.14 | |  | | 52 | | 0.07 | |  | |
| 8–<9 years | | 17 | | 0.09 | |  | | 36 | | 0.05 | |  | |
| 9–<10 years | | 100 | | 0.05 | |  | | 0 | | 0.00 | |  | |
| 10–<11 years | | 97 | | 0.13 | |  | | 72 | | 0.06 | |  | |
| **Index year** | |  | |  | |  | |  | |  | |  | |
| 2005 (REF) | | 205 | | 0.29 | | REF | |  | |  | |  | |
| 2006 | | 118 | | 0.18 | | 0.62 (0.49, 0.78) | |  | |  | |  | |
| 2007 | | 45 | | 0.07 | | 0.25 (0.18, 0.34) | |  | |  | |  | |
| 2008 | |  | |  | |  | | 81 | | 0.20 | | REF | |
| 2009 | |  | |  | |  | | 20 | | 0.05 | | 0.27 (0.17, 0.44) | |
| 2010 | |  | |  | |  | | 52 | | 0.15 | | 0.72 (0.51, 1.02) | |
| 2011 | |  | |  | |  | | 28 | | 0.08 | | 0.40 (0.26, 0.61) | |
| 2012 | |  | |  | |  | | 11 | | 0.04 | | 0.18 (0.1, 0.34) | |
| 2013 | |  | |  | |  | | 0 | | - | | - | |
| 2014 | |  | |  | |  | | 22 | | 0.07 | | 0.34 (0.21, 0.55) | |
| 2015 | |  | |  | |  | | 23 | | 0.05 | | 0.27 (0.17, 0.43) | |
| 2016 | |  | |  | |  | | 20 | | 0.05 | | 0.23 (0.14, 0.38) | |
| 2017 | |  | |  | |  | | 0 | | - | | - | |
| 2018 | |  | |  | |  | | 0 | | - | | - | |
| 2019 | |  | |  | |  | | 27 | | 0.07 | | 0.35 (0.23, 0.54) | |
| 2020 | |  | |  | |  | | 0 | | - | | - | |
| 2021 | |  | |  | |  | | 0 | | - | | - | |
| 2022 | |  | |  | |  | | 0 | | - | | - | |
| **Medical Condition/Medication Use Potentially Increasing IMD Risk^d^** | | | | | | | |  | |  | |  | |
| **Clinical conditions^e^** | |  | |  | |  | |  | |  | |  | |
| Asthma | | 0 | | - | | - | | 27 | | 0.22 | | 4.42 (2.97, 6.57) | |
| Immunodeficiency | | 0 | | - | | - | | 27 | | 15.19 | | 311 (209, 462) | |
| **IMD-related medications^e^** | |  | |  | |  | |  | |  | |  | |
| Corticosteroid use | | 0 | | - | | - | | 27 | | 0.17 | | 3.30 (2.22, 4.91) | |
| Oral | | 0 | | - | | - | | 27 | | 0.18 | | 3.64 (2.45, 5.41) | |
| Inhaled | | 0 | | - | | - | | 27 | | 0.31 | | 6.18 (4.16, 9.19) | |
| Intravenous | | 0 | | - | | - | | 0 | | - | | - | |
| Eculizumab | | 0 | | - | | - | | 0 | | - | | - | |
| Ravulizumab | | 0 | | - | | - | | 0 | | - | | - | |
| **IMD vaccination** | | 0 | | - | | - | | 0 | | - | | - | |
| **Number of medical conditions/medications^f^** | |  | |  | |  | |  | |  | |  | |
| 0 | | 368 | | 0.19 | | REF | | 256 | | 0.05 | | REF | |
| 1 | | 0 | | - | | - | | 0 | | - | | - | |
| 2 | | 0 | | - | | - | | 27 | | 3.40 | | 67.10 (45.1, 99.8) | |
| **Geographic Characteristics^b^** | |  | |  | |  | |  | |  | |  | |
| **Population density** | |  | |  | |  | |  | |  | |  | |
| Urban (REF) | | 184 | | 0.11 | | REF | | 261 | | 0.06 | | REF | |
| Rural | | 184 | | 0.72 | | 6.72 (5.48, 8.24) | | 23 | | 0.04 | | 0.68 (0.44, 1.04) | |
| Unknown | | 0 | | - | | - | | 0 | | - | | - | |
| **Census geographic region^g^** | |  | |  | |  | |  | |  | |  | |
| Northeast | |  | | 0.27 | | REF | |  | | 0.09 | | REF | |
| New England | |  | | 0.56 | | 4.07 (2.79, 5.93) | |  | | - | | - | |
| Middle Atlantic | |  | | 0.14 | | REF | |  | | 0.13 | | REF | |
| North Central | |  | | 0.21 | | 0.76 (0.58, 0.99) | |  | | 0.02 | | 0.23 (0.15, 0.35) | |
| East North Central | |  | | 0.14 | | 1.01 (0.66, 1.55) | |  | | 0.03 | | 0.24 (0.16, 0.37) | |
| West North Central | |  | | 0.32 | | 2.37 (1.59, 3.53) | |  | | - | | - | |
| South | |  | | 0.21 | | 0.77 (0.60, 0.99) | |  | | 0.02 | | 0.21 (0.14, 0.31) | |
| South Atlantic | |  | | 0.28 | | 2.01 (1.38, 2.94) | |  | | 0.01 | | 0.08 (0.04, 0.16) | |
| East South Central | |  | | 0.52 | | 3.79 (2.48, 5.79) | |  | | 0.04 | | 0.34 (0.19, 0.60) | |
| West South Central | |  | | - | | - | |  | | 0.02 | | 0.18 (0.10, 0.33) | |
| West | |  | | 0.07 | | 0.25 (0.17, 0.37) | |  | | 0.11 | | 1.22 (0.93, 1.60) | |
| Mountain | |  | | - | | - | |  | | 0.03 | | 0.22 (0.12, 0.41) | |
| Pacific | |  | | 0.10 | | 0.72 (0.45, 1.14) | |  | | 0.15 | | 1.19 (0.90, 1.57) | |
| **HHS region^g^** | |  | |  | |  | |  | |  | |  | |
| Region 1 | |  | | 0.56 | | REF | |  | | - | | - | |
| Region 2 | |  | | - | | - | |  | | 0.14 | | REF | |
| Region 3 | |  | | 0.41 | | 0.73 (0.54, 0.99) | |  | | 0.04 | | 0.28 (0.17, 0.47) | |
| Region 4 | |  | | 0.27 | | 0.48 (0.35, 0.66) | |  | | 0.02 | | 0.15 (0.09, 0.24) | |
| Region 5 | |  | | 0.13 | | 0.23 (0.16, 0.33) | |  | | 0.03 | | 0.19 (0.12, 0.30) | |
| Region 6 | |  | | - | | - | |  | | 0.02 | | 0.15 (0.08, 0.28) | |
| Region 7 | |  | | 0.40 | | 0.71 (0.50, 1.02) | |  | | - | | - | |
| Region 8 | |  | | - | | - | |  | | 0.06 | | 0.44 (0.24, 0.82) | |
| Region 9 | |  | | 0.06 | | 0.10 (0.06, 0.17) | |  | | 0.11 | | 0.76 (0.55, 1.05) | |
| Region 10 | |  | | 0.19 | | 0.35 (0.20, 0.61) | |  | | 0.15 | | 1.02 (0.69, 1.50) | |

a IR reported per 100,000 PY

b Measured on index date

c Person-time at risk was calculated during each age interval; individuals could contribute person-time to multiple age ranges based on length of continuous enrollment

d Measured during the 6-month baseline period

e For each medical condition/medication use the referent group is individuals without each medical condition/medication use

f Medical conditions/medications include: asplenia, asthma, autoimmune disease, complement component deficiency, hemophilia, HIV infection, hematopoietic stem cell transplant, immunodeficiency, organ transplant, liver disease, malignancy, preterm delivery, renal disease, sickle cell anemia, eculizumab, and ravulizumab

g Number of cases cannot be reported in commercial for regions with <30 cases

Abbreviations: CI: confidence interval; HHS: Health and Human Services; IMD: invasive meningococcal disease; IR: incidence rate; IRR: incidence rate ratio; PY: person-years; REF: reference

Supplemental Table 3. Incidence rate by demographic, clinical, and geographic characteristics, toddler cohorts, 2005–2022

|  | **Commercial**  **N=13,628,626** | | | **Medicaid**  **N=10,730,394** | | |
| --- | --- | --- | --- | --- | --- | --- |
|  | N | IR^a^ | IRR (95% CI) | N | IR^a^ | IRR (95% CI) |
| **Demographic Characteristics^b^** | | | | | | |
| **Sex** |  |  |  |  |  |  |
| Male (REF) | 20 | 0.19 | REF | 41 | 0.45 | REF |
| Female | 14 | 0.14 | 0.73 (0.37, 1.47) | 39 | 0.45 | 1.00 (0.65, 1.56) |
| **Race^c^** |  |  |  |  |  |  |
| White (REF) |  |  |  | 52 | 0.69 | REF |
| Black |  |  |  | 7 | 0.13 | 0.20 (0.09, 0.43) |
| American Indian |  |  |  | 0 | - | - |
| Hispanic |  |  |  | 7 | 0.32 | 0.46 (0.21, 1.01) |
| Asian or Pacific Islands |  |  |  | 2 | 0.70 | 1.02 (0.25, 4.20) |
| Other |  |  |  | 12 | 0.46 | 0.68 (0.36, 1.27) |
| **Age on index** |  |  |  |  |  |  |
| 1–<2 years | 23 | 0.17 | REF | 67 | 0.46 | REF |
| 2–<3 years | 6 | 0.18 | 1.12 (0.46, 2.75) | 3 | 0.17 | 0.36 (0.11, 1.15) |
| 3–<4 years | 3 | 0.13 | 0.77 (0.23, 2.57) | 8 | 0.64 | 1.39 (0.67, 2.90) |
| 4–<5 years | 2 | 0.21 | 1.26 (0.30, 5.36) | 2 | 0.40 | 0.86 (0.21, 3.52) |
| **Index year** |  |  |  |  |  |  |
| 2005 (REF) | 9 | 0.49 | REF | 26 | 0.97 | REF |
| 2006 | 2 | 0.18 | 0.37 (0.08, 1.70) | 10 | 2.02 | 2.09 (1.01, 4.33) |
| 2007 | 4 | 0.34 | 0.71 (0.22, 2.30) | 3 | 0.60 | 0.62 (0.19, 2.06) |
| 2008 | 6 | 0.29 | 0.60 (0.22, 1.70) | 8 | 0.63 | 0.65 (0.29, 1.43) |
| 2009 | 2 | 0.12 | 0.24 (0.05, 1.10) | 2 | 0.20 | 0.20 (0.05, 0.85) |
| 2010 | 1 | 0.06 | 0.13 (0.02, 1.04) | 6 | 0.66 | 0.68 (0.28, 1.65) |
| 2011 | 3 | 0.19 | 0.39 (0.10, 1.43) | 1 | 0.12 | 0.13 (0.02, 0.93) |
| 2012 | 0 | - | - | 6 | 0.40 | 0.41 (0.17, 1.00) |
| 2013 | 2 | 0.17 | 0.34 (0.07, 1.59) | 0 | - | - |
| 2014 | 2 | 0.19 | 0.39 (0.08, 1.79) | 3 | 0.28 | 0.29 (0.09, 0.97) |
| 2015 | 1 | 0.09 | 0.18 (0.02, 1.41) | 4 | 0.33 | 0.34 (0.12, 0.90) |
| 2016 | 1 | 0.11 | 0.22 (0.03, 1.72) | 3 | 0.28 | 0.29 (0.09, 0.97) |
| 2017 | 0 | - | - | 2 | 0.21 | 0.22 (0.05,0.93) |
| 2018 | 0 | - | - | 3 | 0.31 | 0.32 (0.10,1.05) |
| 2019 | 1 | 0.14 | 0.28 (0.04, 2.21) | 1 | 0.09 | 0.09 (0.01,0.69) |
| 2020 | 0 | - | - | 1 | 0.13 | 0.13 (0.02,0.98) |
| 2021 | 0 | - | - | 1 | 0.24 | 0.25 (0.04, 1.84) |
| 2022 | 0 | - | - | 0 | - | - |
| **Medical Condition/Medication Use Potentially Increasing IMD Risk^d^** | | | | | | |
| **Clinical conditions^e^** |  |  |  |  |  |  |
| Asthma | 2 | 0.70 | 4.43 (1.06, 18.47) | 1 | 0.16 | 0.36 (0.05, 2.56) |
| Autoimmune disease | 1 | 0.24 | 1.45 (0.20, 10.57) | 3 | 0.44 | 0.99 (0.31, 3.12) |
| Preterm delivery | 0 | - | - | 1 | 0.49 | 1.10 (0.15, 7.88) |
| Sickle cell anemia | 0 | - | - | 1 | 2.79 | 6.33 (0.88, 45.49) |
| **IMD-related medications^e^** |  |  |  |  |  |  |
| Corticosteroid use | 1 | 0.25 | 1.50 (0.21, 10.96) | 3 | 0.45 | 1.01 (0.32, 3.19) |
| Oral | 3 | 0.43 | 2.76 (0.84, 9.04) | 3 | 0.23 | 0.51 (0.16, 1.61) |
| Inhaled | 1 | 0.42 | 2.60 (0.36, 18.99) | 1 | 0.23 | 0.52 (0.07, 3.71) |
| Intravenous | 0 | - | - | 2 | 0.87 | 1.99 (0.49, 8.10) |
| Other | 0 | - | - | 0 | - | - |
| Eculizumab | 0 | - | - | 0 | - | - |
| Ravulizumab | 0 | - | - | 0 | - | - |
| **Number of medical conditions/medications^f^** |  |  |  |  |  |  |
| 0 | 31 | 0.16 | REF | 74 | 0.45 | REF |
| 1 | 3 | 0.39 | 2.50 (0.76, 8.17) | 6 | 0.45 | 1.00 (0.43, 2.29) |
| 2 | 0 | - | - | 0 | - | - |
| 3+ | 0 | - | - | 0 | - | - |
| **Geographic Characteristics^b^** | | | | | | |
| **Population density** |  |  |  |  |  |  |
| Urban (REF) | 28 | 0.16 | REF | 60 | 0.45 | REF |
| Rural | 6 | 0.24 | 1.53 (0.63, 3.69) | 19 | 0.45 | 1.01 (0.60, 1.69) |
| Unknown | 0 | - | - | 1 | 0.35 | 0.79 (0.11, 5.67) |
| **Census geographic region^g^** |  |  |  |  |  |  |
| Northeast |  | 0.12 | REF |  |  |  |
| New England |  | 0.26 | 3.12 (0.44, 22.16) |  |  |  |
| Middle Atlantic |  | 0.08 | REF |  |  |  |
| North Central |  | 0.25 | 2.03 (0.66, 6.31) |  |  |  |
| East North Central |  | 0.24 | 2.89 (0.61, 13.63) |  |  |  |
| West North Central |  | 0.29 | 3.54 (0.65, 19.31) |  |  |  |
| South |  | 0.12 | 0.99 (0.31, 3.15) |  |  |  |
| South Atlantic |  | 0.10 | 1.18 (0.22, 6.46) |  |  |  |
| East South Central |  | 0.34 | 4.08 (0.75, 22.28) |  |  |  |
| West South Central |  | 0.07 | 0.87 (0.12, 6.14) |  |  |  |
| West |  | 0.20 | 1.59 (0.48, 5.28) |  |  |  |
| Mountain |  | 0.14 | 1.70 (0.24, 11.99) |  |  |  |
| Pacific |  | 0.23 | 2.81 (0.57, 13.93) |  |  |  |
| Unknown |  | - | - |  |  |  |
| **HHS region^g^** |  |  |  |  |  |  |
| Region 1 |  | 0.26 | REF |  |  |  |
| Region 2 |  | 0.06 | 0.24 (0.02, 2.67) |  |  |  |
| Region 3 |  | 0.20 | 0.80 (0.15, 4.34) |  |  |  |
| Region 4 |  | 0.12 | 0.47 (0.09, 2.41) |  |  |  |
| Region 5 |  | 0.22 | 0.86 (0.18, 4.05) |  |  |  |
| Region 6 |  | 0.10 | 0.40 (0.07, 2.38) |  |  |  |
| Region 7 |  | 0.38 | 1.51 (0.27, 8.19) |  |  |  |
| Region 8 |  | 0.15 | 0.58 (0.05, 6.35) |  |  |  |
| Region 9 |  | 0.20 | 0.77 (0.15, 3.99) |  |  |  |
| Region 10 |  | 0.13 | 0.50 (0.05, 5.50) |  |  |  |
| Unknown |  | - | - |  |  |  |

a IR reported per 100,000 PY

b Measured on index date

c Race is not available for the Commercial cohort

d Measured during the 6-month baseline period

e For each condition/medication use the referent group is individuals without each medical condition/medication use

f Medical conditions/medication use include: asplenia, asthma, autoimmune disease, complement component deficiency, hemophilia, HIV infection, hematopoietic stem cell transplant, immunodeficiency, organ transplant, liver disease, malignancy, preterm delivery, renal disease, sickle cell anemia, eculizumab, and ravulizumab

g Region is not reportable for the Medicaid cohort due to data use agreements with MarketScan data contributors; number of cases cannot be reported in commercial for regions with <30 cases

Abbreviations: CI: confidence interval; HHS: Health and Human Services; IMD: invasive meningococcal disease; IR: incidence rate; IRR: incidence rate ratio; PY: person-years; REF: reference

Supplemental Table 4. Incidence rate by demographic, clinical, and geographic characteristics, child cohorts, 2005–2022

|  | **Commercial**  **N=18,506,739** | | | **Medicaid**  **N=9,588,361** | | |
| --- | --- | --- | --- | --- | --- | --- |
|  | N | IR^a^ | IRR (95% CI) | N | IR^a^ | IRR (95% CI) |
| **Demographic Characteristics^b^** | | | | | | |
| **Sex** |  |  |  |  |  |  |
| Male (REF) | 14 | 0.08 | REF | 18 | 0.17 | REF |
| Female | 22 | 0.13 | 1.64 (0.84, 3.20) | 19 | 0.20 | 1.13 (0.59, 2.15) |
| **Race^c^** |  |  |  |  |  |  |
| White (REF) |  |  |  | 18 | 0.21 | REF |
| Black |  |  |  | 14 | 0.24 | 1.15 (0.57, 2.30) |
| American Indian |  |  |  | 1 | 0.76 | 3.69 (0.49, 27.62) |
| Hispanic |  |  |  | 1 | 0.04 | 0.19 (0.02, 1.38) |
| Asian or Pacific Islands |  |  |  | 0 | - | - |
| Other |  |  |  | 3 | 0.13 | 0.66 (0.19, 2.23) |
| **Age on index** |  |  |  |  |  |  |
| 5–<6 years | 14 | 0.08 | REF | 16 | 0.12 | REF |
| 6–<7 years | 5 | 0.11 | 1.47 (0.52, 4.07) | 8 | 0.40 | 3.43 (1.46, 8.01) |
| 7–<8 years | 8 | 0.20 | 2.59 (1.08, 6.17) | 3 | 0.18 | 1.50 (0.43, 5.16) |
| 8–<9 years | 1 | 0.03 | 0.38 (0.05, 2.92) | 6 | 0.43 | 3.66 (1.43, 9.36) |
| 9–<10 years | 5 | 0.20 | 2.65 (0.95, 7.36) | 3 | 0.30 | 2.53 (0.73, 8.67) |
| 10–<11 years | 3 | 0.30 | 3.96 (1.13, 13.76) | 1 | 0.24 | 2.06 (0.27, 15.52) |
| **Index year** |  |  |  |  |  |  |
| 2005 (REF) | 12 | 0.32 | REF | 24 | 0.64 | REF |
| 2006 | 3 | 0.15 | 0.47 (0.13, 1.66) | 2 | 0.35 | 0.54 (0.12, 2.26) |
| 2007 | 2 | 0.10 | 0.32 (0.07, 1.42) | 2 | 0.41 | 0.63 (0.14, 2.67) |
| 2008 | 4 | 0.11 | 0.35 (0.11, 1.08) | 5 | 0.37 | 0.58 (0.22, 1.51) |
| 2009 | 2 | 0.07 | 0.21 (0.04, 0.95) | 1 | 0.10 | 0.16 (0.02, 1.15) |
| 2010 | 4 | 0.16 | 0.49 (0.15, 1.52) | 0 | - | - |
| 2011 | 2 | 0.08 | 0.24 (0.05, 1.07) | 0 | - | - |
| 2012 | 1 | 0.05 | 0.15 (0.01, 1.13) | 2 | 0.10 | 0.15 (0.03, 0.63) |
| 2013 | 0 | - | - | 0 | - | - |
| 2014 | 2 | 0.12 | 0.37 (0.08, 1.66) | 0 | - | - |
| 2015 | 2 | 0.11 | 0.34 (0.07, 1.49) | 0 | - | - |
| 2016 | 1 | 0.07 | 0.21 (0.02, 1.65) | 0 | - | - |
| 2017 | 0 | - | - | 0 | - | - |
| 2018 | 0 | - | - | 0 | - | - |
| 2019 | 1 | 0.10 | 0.31 (0.04, 2.40) | 1 | 0.08 | 0.13 (0.01, 0.94) |
| 2020 | 0 | - | - | 0 | - | - |
| 2021 | 0 | - | - | 0 | - | - |
| 2022 | 0 | - | - | 0 | - | - |
| **Medical Condition/Medication Use Potentially Increasing IMD Risk^d^** | | | |  |  |  |
| **Clinical conditions^e^** |  |  |  |  |  |  |
| Asplenia | 0 | - | - | 0 | - | - |
| Asthma | 1 | 0.15 | 1.47 (0.20, 10.72) | 1 | 0.11 | 0.60 (0.08, 4.40) |
| Immunocompromised | 1 | 13.71 | 133.08 (18.23, 971.40) | 0 | - | - |
| Sickle cell anemia | 0 | - | - | 1 | 4.77 | 26.56 (3.64, 193.69) |
| **IMD-related medications^e^** |  |  |  |  |  |  |
| Corticosteroid use | 1 | 0.12 | 1.10 (0.15, 8.05) | 0 | - | - |
| Oral | 1 | 0.13 | 1.27 (0.17, 9.30) | 1 | 0.11 | 0.60 (0.08, 4.41) |
| Inhaled | 1 | 0.22 | 2.07 (0.28, 15.07) | 0 | - | - |
| Intravenous | 0 | - | - | 0 | - | - |
| Other | 0 | - | - | 0 | - | - |
| Eculizumab | 0 | - | - | 0 | - | - |
| Ravulizumab | 0 | - | - | 0 | - | - |
| **Number of medical conditions/medications^f^** |  |  |  |  |  |  |
| 0 | 35 | 0.11 | REF | 35 | 0.19 | REF |
| 1 | 0 | - | - | 2 | 0.17 | 0.93 (0.22, 3.85) |
| 2 | 1 | 2.38 | 22.40 (3.07, 164) | 0 | - | - |
| 3+ | 0 | - | - | 0 | - | - |
| **Geographic Characteristics^b^** |  |  |  |  |  |  |
| **Population density** |  |  |  |  |  |  |
| Urban | 27 | 0.04 | REF | 29 | 0.19 | REF |
| Rural | 9 | 0.10 | 2.20 (1.04, 4.69) | 8 | 0.17 | 0.86 (0.39, 1.88) |
| Unknown | 0 | - | - | 0 | - | - |
| **Census geographic region^g^** |  |  |  |  |  |  |
| Northeast |  | 0.08 | REF |  |  |  |
| New England |  | 0.08 | 0.88 (0.18, 4.22) |  |  |  |
| Middle Atlantic |  | 0.08 | REF |  |  |  |
| North Central |  | 0.04 | 0.53 (0.20, 1.41) |  |  |  |
| East North Central |  | 0.04 | 0.50 (0.16, 1.59) |  |  |  |
| West North Central |  | 0.04 | 0.53 (0.11, 2.51) |  |  |  |
| South |  | 0.04 | 0.48 (0.20, 1.16) |  |  |  |
| South Atlantic |  | 0.04 | 0.50 (0.17, 1.49) |  |  |  |
| East South Central |  | 0.10 | 1.12 (0.33, 3.82) |  |  |  |
| West South Central |  | 0.01 | 0.12 (0.02, 1.00) |  |  |  |
| West |  | 0.07 | 0.81 (0.32, 2.04) |  |  |  |
| Mountain |  | 0.02 | 0.24 (0.03, 1.98) |  |  |  |
| Pacific |  | 0.09 | 1.10 (0.39, 2.99) |  |  |  |
| Unknown |  | - | - |  |  |  |
| **HHS region^g^** |  |  |  |  |  |  |
| Region 1 |  | 0.08 | REF |  |  |  |
| Region 2 |  | 0.08 | 0.99 (0.18, 5.41) |  |  |  |
| Region 3 |  | 0.09 | 1.22 (0.25, 6.06) |  |  |  |
| Region 4 |  | 0.05 | 0.65 (0.13, 3.11) |  |  |  |
| Region 5 |  | 0.04 | 0.54 (0.10, 2.77) |  |  |  |
| Region 6 |  | 0.01 | 0.13 (0.01, 1.48) |  |  |  |
| Region 7 |  | 0.05 | 0.77 (0.11, 5.46) |  |  |  |
| Region 8 |  | 0.04 | 0.61 (0.06, 6.76) |  |  |  |
| Region 9 |  | 0.05 | 0.64 (0.12, 3.51) |  |  |  |
| Region 10 |  | 0.15 | 1.97 (0.36, 10.73) |  |  |  |
| Unknown |  | - | - |  |  |  |

a IR reported per 100,000 PY

b Measured on index date

c Race is not available for the Commercial cohort

d Measured during the 6-month baseline period

e For each condition/medication use the referent group is individuals without each medical condition/medication use

f Medical conditions/medication use include: asplenia, asthma, autoimmune disease, complement component deficiency, hemophilia, HIV infection, hematopoietic stem cell transplant, immunodeficiency, organ transplant, liver disease, malignancy, preterm delivery, renal disease, sickle cell anemia, eculizumab, and ravulizumab

g Region is not reportable for the Medicaid cohort due to data use agreements with MarketScan data contributors; number of cases cannot be reported in commercial for regions with <30 cases

Abbreviations: CI: confidence interval; HHS: Health and Human Services; IMD: invasive meningococcal disease; IR: incidence rate; IRR: incidence rate ratio; PY: person-years; REF: reference
